# Rapid Identification between Two Fish Species Using UV-Vis Spectroscopy for Substitution Detection

**DOI:** 10.3390/molecules26216529

**Published:** 2021-10-28

**Authors:** Zhaoliang Chai, Chengyu Wang, Hongyan Bi

**Affiliations:** College of Food Science and Engineering, Shanghai Ocean University, Shanghai 201306, China; chaizhaoliang2020@163.com (Z.C.); wangchengyu1002@163.com (C.W.)

**Keywords:** UV-Vis spectroscopy, fish substitution, trifluoroacetic acid (TFA), principal component analysis (PCA)

## Abstract

Fish species substitution and fraud has become a worldwide economic issue in the seafood industry. In this study, an ultraviolet-visible (UV-Vis) spectroscopy-based method was developed for the identification of fish samples. Sixty fish samples from twelve commonly consumed fish species in China were analyzed as models to testify the protocol. The obtained results show that UV-Vis spectroscopy combined with chemometric analysis, such as principal component analysis (PCA), can accurately distinguish two fish species by boiling fish tissue sample in trifluoroacetic acid (TFA) solution for 2 min and analyzing the resultant samples using a UV-Vis spectrometer. The developed strategy was successfully applied to the classification and identification of fish samples on the market. It is a promising strategy that can be applied to the classification and authenticity testing of closely related fish species in order to detect and recognize fish substitution.

## 1. Introduction

Fighting food fraud is challenging because of the constant evolution of fraudulent practices and its implications on both consumers and the globalized trade [1,2,3]. Fish is a vulnerable commodity for fraud, owing to the high national production, importation and consumption. It is important to monitor the entire food chain in order to detect and prevent fraudulent actions, such as species substitutions, which is considered the most common fraud in the seafood industry [4]. Rapid and easy methods for food authenticity tests are always necessary with the developments in globalization of the food supply chain.

Spectroscopic techniques, including vibrational (near-infrared (NIR), mid-infrared (MIR), Raman), fluorescence or absorption ultraviolet-visible (UV-Vis), and nuclear magnetic resonance (NMR) spectroscopies, together with hyperspectral imaging (HSI) spectroscopy, have been used to develop tools for food quality analysis and authenticity testing [5]. Puertas et al. determined the cholesterol in egg yolks using Ultraviolet-Visible-near-IR Spectroscopy (UV-VIS-NIR) [6]. Plasticizer diethylhexyl phthalate in drinks was determined using diffuse reflectance UV spectroscopy coupled with membrane filtration [7]. We previously developed microfluidic bio-sensors integrated with UV-Vis spectrometry for specific analysis in samples with complex matrices such as human serum and alimentary food products [8,9]. Minced beef adulteration with turkey meat was detected using UV-Vis, NIR and MIR spectroscopy [10]. UV-Vis spectroscopy and attenuated total reflection-Fourier transform infrared (ATR-FTIR) spectroscopy coupled with high-performance liquid chromatography (HPLC) methods were used to detect food fraud in commercial pomegranate molasses syrups [11]. Pork adulterated with beef and mutton was identified using infrared spectroscopy [12]. Chinese vinegar samples were classified via volatiles using long-optical-path infrared spectroscopy and chemometrics [13]. NIR spectroscopy and chemometrics were applied for the typification of Spanish wine vinegars with a protected designation of origin [14]. Olive oils from the Mediterranean region were evaluated using UV-Vis spectroscopy and independent component analysis [15]. Raman chemical imaging method has been developed to authenticate skim milk powder where identification and distribution of the multiple adulterant particles in the milk powder could be visualized using Raman chemical images [16].

Spectroscopic strategies have been developed for the analysis of seafood authenticity. The authenticity issues of fish and seafood products involve species substitution, geographical origin falsification, production method or farming system misrepresentation and fresh for frozen/thawed product substitution. Qualitative spectroscopy and chemometrics used in seafood products were summarized by Ghidini et al. in 2019 [17]. Near-infrared diffuse reflectance spectroscopy was utilized to recognize marine fish surimi [18]. Velioğlu et al. used Raman spectroscopy coupled with chemometrics to differentiate fresh and frozen-thawed fish samples, where 64 fish samples from 6 different species, namely horse mackerel (*Trachurus trachurus*), European anchovy (*Engraulis encrasicolus*), red mullet (*Mullus surmuletus*), bluefish (*Pomatamus saltatrix*), Atlantic salmon (*Salmo salar*) and flying gurnard (*Trigla lucerna*) were analyzed [19]. We recently investigated the storage situation of fish muscle via the analysis of its exudate by matrix-assisted laser desorption/ionization time-of-flight mass spectrometry (MALDI-TOF MS), where the changes of exudates of fish muscle with different freeze-thaw cycles (0, 1 and 2) and frozen storage periods were compared [20]. We also studied fish samples for their classification, authentication and taxonomy via mass spectrometric, metabolomic and chemometric methods in the recent years [21,22,23]. The pretreatment of the fish muscle samples was taken into account to enhance the analysis efficiency [24]. However, there are no reports based on UV-Vis spectroscopy for fish classification and authentication to the best knowledge of the authors.

The objective of this study was to obtain an easy and efficient method for the recognition of commercial fish species and the detection of fish substitution based on UV-Vis spectroscopy. Experimentally, fish muscle tissue from the fish sample was used to obtain extracts for performing UV-Vis spectroscopic analysis. UV-Vis spectroscopy combined with the chemometric method was used to analyze the extracts of different fishes. Twelve fish species including nine marine fishes and three freshwater fishes were taken as models to test the protocol. Fish samples from the market were identified, and genetic testing was utilized to validate the results. This method is the first to classify and identify fish species using UV-Vis spectroscopy. It is a promising strategy that can be utilized for rapid authenticity testing of suspicious fish samples, especially those with a high value.

## 2. Results and Discussion

### 2.1. Impact of Dilution on Classification of UV-Vis Spectra of Different Fish Samples

Trifluoroacetic acid (TFA) solution was utilized to extract proteins from samples of fish muscle for the discovery of species-specific biomarkers using matrix-assisted laser desorption/ionization time-of-flight mass spectrometry (MALDI-TOF MS) [25]. We previously evaluated that the TFA acid method is optimal and capable of pretreating fish muscle tissue for seafood analysis, as a reproducible and efficient method of producing good spectral resolution when analyzing the resultant samples using MALDI-TOF MS [24]. 

**Figure 1:**
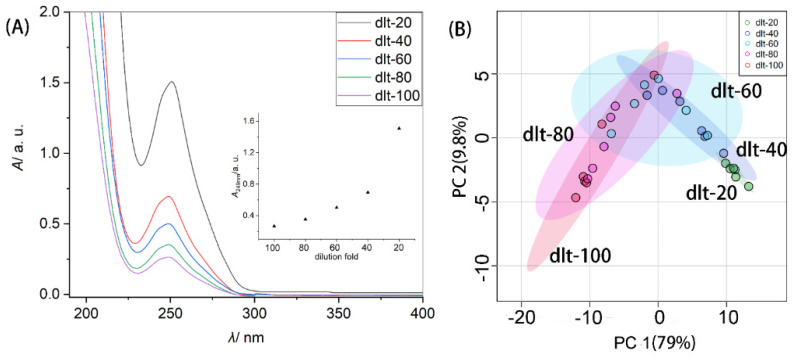
(**A**) UV-Vis spectra of extracts from 2 g of fish fillet sample of *Scomberomorus niphonius* (SN) with dilution folds of 20, 40, 60, 80 and 100 with deionized (DI) water. Inset is the plot of absorbance of solution of muscle extract at 249 nm versus dilution fold. (**B**) Plot of principal component analysis (PCA) scores for the UV-Vis spectroscopic results of tissue extracts of different concentrations of extract from fish, SN. The extraction was conducted following the protocol detailed in experimental Section 3.2. That is, the initial extract of fish muscle sample was obtained by pre-treating 2 g of fish muscle tissue sample with 10 mL of trifluoroacetic acid (TFA) aqueous solution (0.1%, *v*/*v*), and diluted 20, 40, 60, 80 and 100-fold respectively with DI water for UV-Vis spectroscopic analysis.

To obtain an appropriate solution for the analysis of fish samples, the fish species Japanese seerfish (*Scomberomorus niphonius*, SN) was taken as a model for extracting bio-materials from 2 g of SN muscle tissue sample with 10 mL of TFA solution (0.1%, *v*/*v*) to perform UV-Vis spectroscopic analysis. UV-Vis spectra of the solution of extracts diluted with different folds were recorded. Figure 1A displays the representative UV-Vis spectra of obtained extracts of fish muscle tissue sample of SN. It can be seen that UV-Vis spectra show maximum absorption at wavelength 249 ± 1 nm. The dilution can change the concentration of extracts, as shown in the inset of Figure 1A. A deviation occurs at high concentrations since there is interaction between absorbing particles at high concentrations, and the absorption characteristics of the sample are affected.

Principal component analysis (PCA) is a classical multivariate statistical technique used for dimension reduction and can assist in revealing the similarity and difference of the target data and visualize the aggregation and dispersion of the data of different samples [26]. To evaluate the impact of dilution on the classification of extracts of fish muscle tissue, UV-Vis spectra of extracts of SN were further analyzed by PCA. Muscle from different parts of fish sample was compared. As shown in Supplementary Material S1, when sampling the muscle from different parts of a fish sample, the UV-Vis spectroscopic analysis is not supposed to be impacted. During the experiment, fish muscle tissue samples from the dorsal or ventral of fish were selected for analysis.

Supplementary Material S2 compares the impact of dilution on the classification of different fish samples. By comparing the plot of PCA scores for UV-Vis spectroscopic datasets of fish muscle extracts under different dilutions, it was found that an 80-fold dilution of the extract obtained from 2 g of muscle tissue sample with 10 mL of TFA solution (0.1 %, *v*/*v*), is appropriate. An 80-fold dilution was made to the obtained fish tissue extract to aid the classification of different fish samples. Figure 1B shows that the concentration of protein content may be an important aspect in distinguishing different fish products. 

### 2.2. Reproducibility of the Utilized Pretreatment Method

Reproducibility is important for estimating uncertainty in a measurement and measuring the sensitivity of a method to laboratory changes. To verify the reproducibility of the presently utilized pretreatment method of the fish analysis, three independent analysis of model fish samples, including large yellow croaker (*Larimichthys crocea*, shortened to LC in the present study), small yellow croaker (*Larimichthys polyactis*, LP), pacific chub mackerel (*Scomber japonicas*, SJ), Japanese seerfish (*Scomberomorus niphonius*, SN), salmon (*Salmo salar*, SS), and rainbow trout (*Oncorhynchus mykiss*, OM) were performed. During the experiment, around 2 g of fish muscle tissue samples were homogenized in 10 mL of 0.1% (*v*/*v*) TFA solution for 2 min, heated at 80 °C for 2 min and centrifuged at 20,000× *g* for 5 min to obtain an initial fish protein extract solution. Table 1 lists the maximum absorption (*A*) at a characteristic wavelength (*λ*_max_) in three different runs of experiments, illustrating the excellent reproducibility of the present protocol for the pretreatment of fish muscle tissues indicated by the less than 6% of relative standard deviations (RSD) values. 

Figure S4 in Supplementary Material S3 compares the plots of the PCA scores for the UV-Vis spectra of analyzed fish samples. It can be observed on the plot of PCA scores that the points representing UV-Vis spectra of one fish species collected in three experiments, clustered in very close areas, indicating similarity among the samples. Experimentally, it is necessary to choose an identical pretreatment for each sample, such as heating temperature and dilution folds. Distinguishing different species of fish via UV-Vis spectroscopic analysis when the pretreatment conditions of fish muscle samples are consistent is a promising strategy.

### 2.3. Testing the UV-Vis Technique in Fish Species Classification

**Figure 2:**
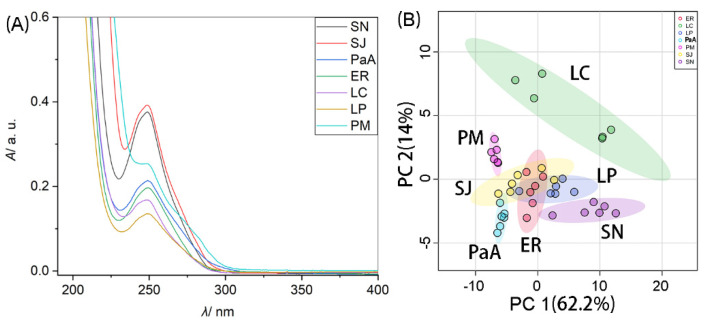
(**A**) UV-Vis spectra of muscle extracts of seven fish species from fish samples of *Scomber japonicus* (SJ), *Scomberomorus niphonius* (SN), *Larimichthys crocea* (LC), *Larimichthys polyactis* (LP), *Epinephelus rivulatus* (ER), *Pampus argenteus* (PaA) and *Pagrosomus major* (PM). (**B**) Plot of principal component analysis (PCA) scores for the UV-Vis spectra of the seven fish species. The other conditions make reference to the information listed in Table 1.

To distinguish fish species based on UV-Vis spectroscopy, seven fish species were selected and analyzed. Figure 2 compares the UV-Vis spectra of the analyzed fish species. It is barely possible to distinguish different fish species by directly comparing their UV-Vis spectra. It can be observed from Figure 2A that under identical pretreatment conditions, different fish samples may contain different content of proteins, indicated by the different maximum absorbance at a characteristic wavelength. This may be a very important aspect of differentiating different fish samples. 

PCA was executed on the obtained UV-Vis spectra of the analyzed fish samples shown in Figure 2A. Figure 2B shows the plot of PCA score for the UV-Vis spectra of the analyzed seven fish species. The color-covered ellipse displays 95% of confidence region. It can be observed that the areas for some fish species cluster in different areas, such as LC, PM, SN and PaA. However, it is difficult to distinguish some fish species, such as SJ, ER and LP.

### 2.4. Demonstration of Applicability of UV-Vis Spectroscopy in Fish Species Authenticity

**Figure 3:**
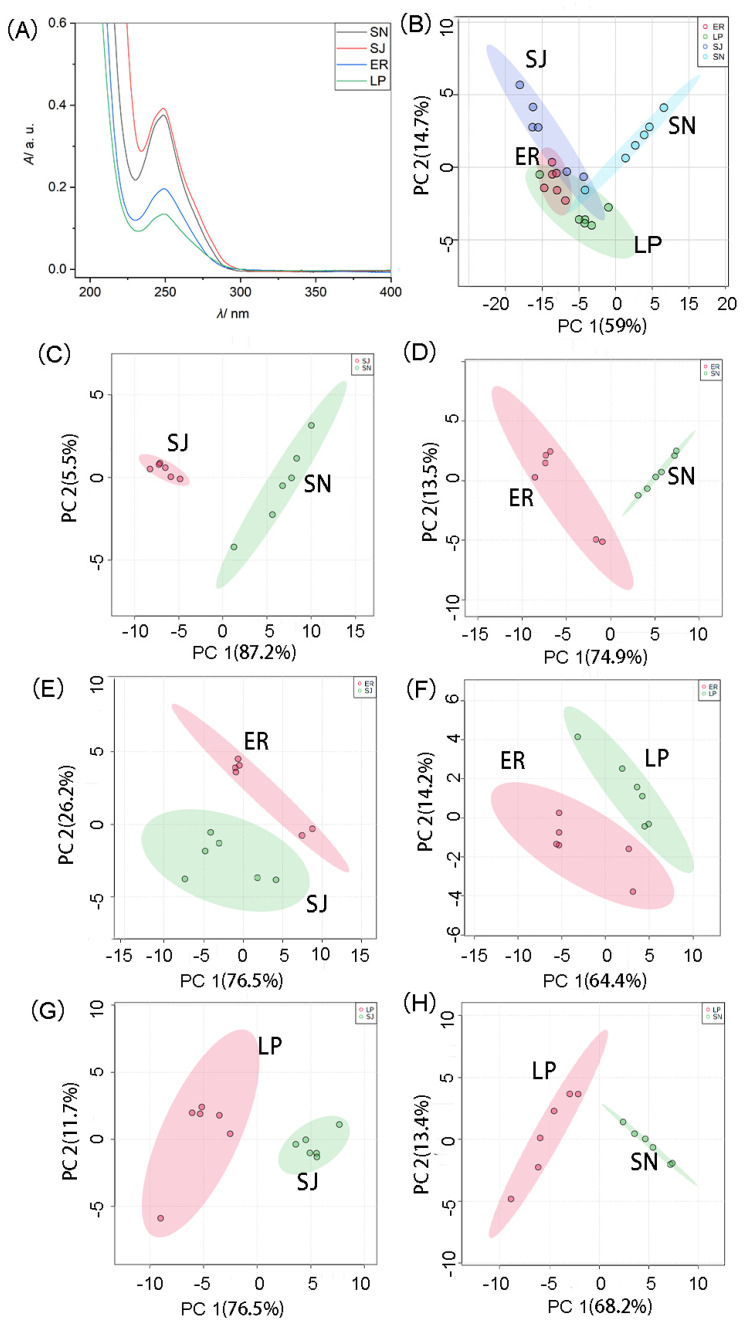
(**A**) UV-Vis spectra of extracts from four fish species samples of *Scomber japonicus* (SJ), *Scomberomorus niphonius* (SN), *Epinephelus rivulatus* (ER) and *Larimichthys polyactis* (LP). (**B**) Plot of principal component analysis (PCA) scores for the UV-Vis spectra of the analyzed fish samples namely fish samples from the species SJ, SN, ER and LP. (**C**–**H**) Plots of PCA scores for the UV-Vis spectra of the analyzed fish samples belonging to any two fish species of SJ, SN, ER and LP. Six UV-Vis spectra were obtained from each fish species. The initial extract was diluted 80-fold with deionized (DI) water. The other conditions make reference to the information listed in experimental Section 3.2.

To further verify the possibility of distinguishing fish samples based on UV-Vis spectroscopy, the UV-Vis spectra of fewer fish species were compared. Figure 3A,B show the UV-Vis spectra of *Scomber japonicus* (SJ), *Scomberomorus niphonius* (SN), *Larimichthys polyactis* (LP) and *Epinephelus rivulatus* (ER), and the PCA score plot for the UV-Vis spectra of the four species, respectively. It can be seen that the four analyzed fish species cannot be differentiated. However, as shown in Figure 3C–H, the points for representing the UV-Vis spectral information of any two fish species of the four analyzed fish species cluster in different areas, illustrating that any two fish species from the analyzed fish samples can clearly be distinguished. 

### 2.5. Distinguishing of Fish Samples at Different Level

To verify the differentiation of fish species via UV-Vis spectroscopy, eight fish species belonging to different levels were analyzed. Supplementary Material S4 compares the plot of PCA scores of two fish species analyzed in the present study. The results show that significant differences exist between the UV-Vis spectra of any two fish species of fish samples analyzed in the present study. It can be concluded that when the number of analyzed fish samples is narrowed down to two, the fish samples can be accurately classified. The results show that fishes belonging to different species level can still be classified. It can be conservatively concluded that any two randomly chosen fish species can be potentially distinguished. 

The present study aimed to develop a method to estimate the prevalence of fish species substitutions. Supplementary Material S4 displays the classification of other fish samples at different levels, showing that two fish samples can be distinguished accurately. 

Two fish species from different levels, including class, order, family and genus were tested with the models, as listed in Table 2. 

**Figure 4:**
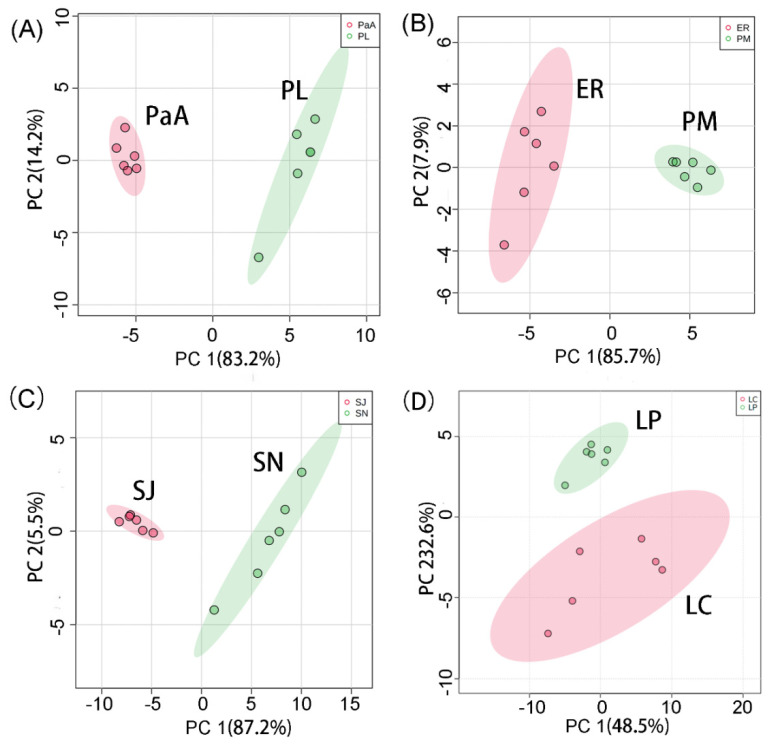
(**A**) Plot of principal component analysis (PCA) scores of UV-Vis spectra of *Pampus argenteus* (PaA) and *Pseudaspius leptocephalus* (PL). PaA and PL both belong to the class Actinopterygii. (**B**) Plot of PCA scores of UV-Vis spectra of *Epinephelus rivulatus* (ER) and *Pagrosomus major* (PM). ER and PM both belong to the order Perciformes. (**C**) PCA score plot of UV-Vis spectra of *Scomberomorus niphonius* (SN) and *Scomber japonicus* (SJ). SN and SJ both belong to the family Scombridae. (**D**) PCA score plot for the UV-Vis spectra of *Larimichthys polyactis* (LP) and *Larimichthys crocea* (LC). LP and LC both belong to the genus Larimichthys. Six UV-Vis spectra were obtained from each fish species. The initial extract was diluted 80-fold with deionized (DI) water for further analysis. The other conditions make reference to the information listed in experimental Section 3.2.

*Pampus argenteus* (PaA) and *Pseudaspius leptocephalus* (PL) both belong to the same class, Actinopterygii. Figure 4A shows the PCA score plot for the UV-Vis spectra of PaA and PL.

*Epinephelus rivulatus* (ER) and *Pagrosomus major* (PM) both belong to the order Perciformes. Figure 4B shows the PCA score plot for the UV-Vis spectra of ER and PM. 

*Scomberomorus niphonius* (SN) and *Scomber japonicus* (SJ) both belong to the family Scombridae. Figure 4C shows the PCA score plot for the UV-Vis spectra of SN and SJ. 

*Larimichthys polyactis* (LP) and *Larimichthys crocea* (LC) both belong to the genus Larimichthys. Figure 4D shows the PCA score plot for the UV-Vis spectra of LP and LC.

The findings show that fishes at the species level can still be distinguished by the present protocol. Applying the present protocol in the classification of two fish samples and the detection of fish substitution is a promising use of the method. 

### 2.6. Classification of Fish Sample on the Market

*Oncorhynchus mykiss* (OM) and *Salmo salar* (SS) both belong to the same family, Salmonidae. Financial loss and health risk, caused by the substitution of *Salmo salar* (SS) with *Oncorhynchus mykiss* (OM), has been widely reported, highlighting the necessity of establishing rapid and accurate methods for their identification and classification. The adulteration of different amounts of *O. mykiss* with *S. salar* was detected by duplex real-time PCR combined with melting curve analysis [27]. Hydrophilic interaction chromatography and mass spectrometry (MS) were used to study the lipidomics of *Oncorhynchus mykiss* and salmon (*Oncorhynchus tshawytscha* and *Salmo salar*), showing the phospholipids among them are significantly different [28].

**Figure 5:**
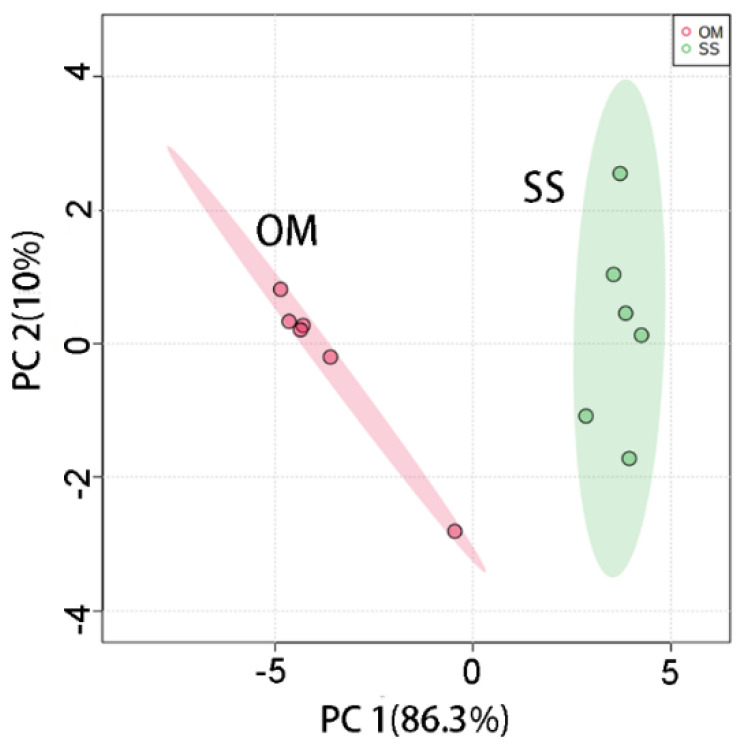
PCA score plot of UV-Vis spectra of *Oncorhynchus mykiss* (OM) and *Salmo salar* (SS). Six UV-Vis spectra were obtained from each fish species. During the experiment, three slices of *Salmo salar* samples were analyzed. Three OM fish samples were analyzed. The initially obtained extracts were diluted 80-fold with deionized (DI) water for further analysis.

Herein, fish samples of OM and fillet of SS were chosen as models to test the feasibility of the present strategy for the classification between them. Figure 5 shows the plot of PCA scores for the UV-Vis spectra of these two fish species. The results show that the present assay allows a fast and accurate classification of SS and OM in processed fish products such as fillets. 

### 2.7. Identification of Incorrectly Labeled Fish Sample for Substitution Detection

Some fishes belonging to Sciaenidae, a family of mainly tropical and subtropical marine percoid fishes that include drums, grunts, and croakers, were used as models to test the feasibility of the present protocol for the detection of fish substitution or fraud. Yellow croaker is a fish with high commercial value in China due to its high meat quality. It has been exploited heavily beyond its ecological resiliency in the recent years, and these stocks have not yet recovered to their former abundance. The scarcity of this species that is mostly appreciated by consumers has led to an increase in their value, commanding a higher market price [29,30]. The high price and limited supply of yellow croaker have resulted in increases in the vulnerability of threat to its populations and the occurrence of fraudulent substitution and mislabeling [31]. 

*Larimichthys crocea* (LC), *Larimichthys polyactis* (LP) and *Pennahia argentata* (PeA) are benthopelagic fish species belonging to the family Sciaenidae. LC, LP and PeA are important economic fish species in China. Fresh specimens of these fish samples can be distinguished by body color, though the colors fade after death, making it possible for these species to be substituted for each other intentionally for financial profit or unintentionally [32]. 

**Figure 6:**
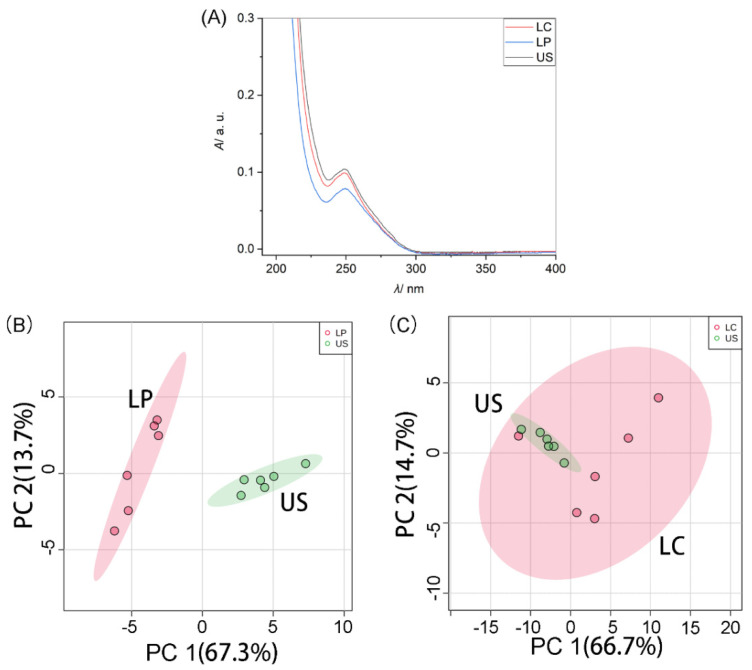
(**A**) UV-Vis spectra in the range 190–400 nm of extracts from the fish samples of *Larimichthys crocea* (LC), *Larimichthys polyactis* (LP) and unknown sample (US). (**B**) Plot of principal component analysis (PCA) scores for the UV-Vis spectra of fish species of LP and US. (**C**) Plot of PCA scores for the UV-Vis spectra of fish species of LC and US. The color-covered area displays 95% of confidence region. Six UV-Vis spectra were obtained from each species. The initial extract was diluted 80-fold for further analysis.

A sample that was claimed to be silver croaker (*Pennahia argentata*, PeA) was bought from the market and analyzed as a model of unknown sample. Figure 6A shows the UV-Vis spectra of *Larimichthys crocea* (LC), *Larimichthys polyactis* (LP) and this unknown sample (US). Figure 6B shows the PCA score plot for the UV-Vis spectra of LP and US. Figure 6C shows the PCA score plot for the UV-Vis spectra of LC and US. The points for the UV-vis spectra of US and LP on the plot of PCA scores do not overlap at all, showing the sample of US does not belong to fish species LP. Figure 6C shows the PCA score plot for the UV-Vis spectra of LC and US. The points on the plot of PCA scores of US and LC cluster in the overlapped ellipse, showing the fish sample US belongs to the fish species LC with a 95% confidence level, since the color-covered area, as shown in Figure 6C in the plot of PCA scores, displays 95% of confidence region.

Genetic testing was performed to authenticate the obtained fish sample. As shown in Figure S9, the unknown sample is genetically identical with the fish species *Larimichthys crocea*, with a confidence level more than 98%. As shown in Figure S10, the obtained *Larimichthys polyactis* sample is genetically identical with the fish species *Larimichthys polyactis*, with a confidence level of more than 97%, illustrating that the obtained fish samples can be certified as *Larimichthys polyactis*. The result obtained by the presently developed method is consistent with the one from the genetic test (Supplementary Material S5). It can be found that the obtained fish was incorrectly labeled or substituted. The findings suggest that the novel approach can assist to identify the substitution of samples with similar morphological features. It can be concluded that the present strategy can be applied to the detection of fish substitution. 

## 3. Materials and Methods

### 3.1. Chemicals and Fish Samples

Trifluoroacetic acid (TFA, ≥99.9%) was purchased from Fisher Scientific Inc. (Loughborough, UK). The DNA kit (HiPure Tissue DNA Mini Kit) was bought from Magen Biotech Co., Ltd. (Guangzhou, Guangdong, China). Deionized (DI) water was obtained by purifying ultra-pure water on a Milli-Q deionized water system (0.22 µm, Millipak Express 40, Burlington, MA, USA) and was used for all the aqueous solutions. 

The fish samples, including pacific chub mackerel (*Scomber japonicas*, shortened as SJ in the present study), Japanese seerfish (*Scomberomorus niphonius*, SN), rainbow trout (*Oncorhynchus mykiss*, OM), large yellow croaker (*Larimichthys crocea*, LC), small yellow croaker (*Larimichthys polyactis*, LP), topmouth culter (*Culter alburnus*, CA), flathead asp (*Pseudaspius leptocephalus*, PL), halfmoon grouper (*Epinephelus rivulatus*, ER), silvery pomfret (*Pampus argenteus*, PaA), red sea bream (*Pagrosomus major*, PM) and silver croaker (*Pennahia argentata*, PeA) were purchased in 2020 from Taobao (China). Three slices of Atlantic salmon (*Salmo salar*, SS) were bought in October 2020 from Ftabest free trade import food center (Pudong New Area, Shanghai, China). Supplementary Material S6 lists the information including the scientific classification, trader, probable origin, size and weight of the fish samples analyzed in the present study. All the fish samples were certified by genetic testing and/or by referring to *fishbase.org*. For the majority of the fish species, six samples were collected, while for ER and OM three samples were collected, and for SS, three slices of fish samples were collected; all of them were used as bio-replicates to represent the corresponding fish species. A fish fillet sample for a single blind analysis was from six fish samples which were obtained in October 2020 from Shili (Ten Mile) seafood store (Taobao, Guangdong, China). All dead fish samples were placed on an ice bag and delivered to the lab. The muscle tissues were cut into manageable portions with a scalpel and stored at −20 °C in sealed bags and used within 2 months to guarantee the quality of samples. Frozen fish samples were thawed overnight at 4 °C prior to further analysis. 

### 3.2. Preparation and UV-Vis Detection of Extract of Fish Muscle Samples

Two grams of fish muscle tissue sample and 10 mL of TFA aqueous solution (0.1%, *v*/*v*) were put in a beaker, mixed thoroughly with a glass rod and homogenized for 2 min by using a homogenizer (F6/F10, Shanghai Jingxin Technology Co., Ltd., Shanghai, China). The mixture was then placed on a hot plate (RH digital, IKA, Staufen, Germany), and heated at 80 °C for 2 min. The resultant liquid was cooled to room temperature (25 °C for about 10 min) and centrifuged at 20,000× *g* for 5 min on a centrifuge system (Centrifuge 5424, Eppendorf, Hamburg, Germany), and then filtered by using 0.22 μm filter membrane. The obtained extract of muscle tissue was diluted 80-fold with DI water for further UV-Vis spectroscopic analysis. The UV-Vis spectrum was recorded at an ambient temperature with a UV-Vis spectrometer (UV-1900 UV-VIS-NIR Spectrophotometer, Shimadzu^®^, Kyoto, Japan). The UV-Vis spectra obtained from six fish samples were collected as a dataset for representing the information of corresponding fish species. In detail, the muscle sample of each fish was pretreated and analyzed to obtain at least one UV-Vis spectrum. When three fish samples were collected, the experiment was repeated twice for each sample to obtain six UV-Vis spectroscopic data.

### 3.3. Genetic Test of Fish Samples 

A sample that was claimed to be silver croaker (*Pennahia argentata*) was obtained from a Taobao online shop (Ten Mile Seafood, Guangdong, China) as a model of unknown fish samples. Genetic testing of the fish samples was performed for their certification by Suzhou Jinwei Biotech Co., Ltd. (Suzhou, Jiangsu, China). In detail, DNA extraction was performed according to the instruction manual of the DNA kit. Universal forward and reverse primers were designed and synthesized with sequences as 5′TCGACTAATCATAAGATATCGGCAC 3′ and 5′ ACTTCAGGGTGACCGAAGAATCAGAA 3′, respectively, for the certification of fish samples belonging to the family Sciaenidae [32]. The reaction systems and conditions for performing polymerase chain reaction (PCR) are listed in Supplementary Material S5. Electrophoresis analysis was performed to identify whether PCR amplifies the expected primers. The PCR products were digested by enzymes for gene sequence test.

### 3.4. Principal Component Analysis (PCA) of the UV-Vis Spectral Data

MetaboAnalyst online software (https://www.metaboanalyst.ca/, accessed on 18 October 2021) was used to perform chemometric analysis, including principal component analysis (PCA), of the obtained UV-Vis spectra.

## 4. Conclusions

Nowadays, fish species substitution and fraud has become an unneglectable concern in the seafood industry. The need for reliable and rapid tests to identify commercial fish is growing. This present study develops a UV-Vis spectroscopic strategy to distinguish the fish species for the detection of substitution and fraud on the seafood market. Fish muscle tissue of the fish sample was used to obtain extracts for performing UV-Vis spectroscopic analysis. The results show that two fish species can be classified by UV-Vis spectroscopy combined with chemometrics method. Twelve fish species were taken as models and analyzed to demonstrate the protocol. Two fish samples on the market can be clearly classified. A fish sample from the market was successfully identified and shown to have an identical result to the one obtained by genetic testing. This method is the first to classify and identify the fish species by UV-Vis spectroscopy. It is promising to utilize the present strategy for rapid authenticity testing of suspicious fish samples, especially those with high value.

## Figures and Tables

**Table 1:** Reproducibility of the pretreatment method. The maximum absorption absorbance (A) at a characteristic wavelength (λ_max_) is listed as mode of average ± standard deviation (SD). Relative standard deviations (RSD) are calculated and listed. Six UV-Vis spectra were obtained from each fish species. The extraction was conducted following the protocol detailed in experimental Section 3.2. Briefly, the initial extract of fish muscle sample was obtained by pretreating 2 g of fish muscle tissue sample with 10 mL of trifluoroacetic acid (TFA) aqueous solution (0.1%, *v*/*v*), and diluted 80-fold with deionized (DI) water for further analysis. Fish samples of *Larimichthys crocea* (LC), *Larimichthys polyactis* (LP), *Scomber japonicas* (SJ), *Scomberomorus niphonius* (SN), *Salmo salar* (SS) and *Oncorhynchus mykiss* (OM) were analyzed.

Run		LC	LP	SJ	SN	SS	OM
**1**	*λ*_max_/nm	249 ± 0.5	249 ± 0.5	248 ± 1.0	248 ± 1.0	249 ± 0.5	249 ± 0.5
	*A*/a. u.	0.111 ± 0.003	0.110 ± 0.005	0.393 ± 0.005	0.361 ± 0.008	0.181 ± 0.005	0.204 ± 0.006
**2**	*λ*_max_/nm	249 ± 0.5	249 ± 0.5	248 ± 1.0	248 ± 1.0	249 ± 0.5	249 ± 0.5
	*A*/a. u.	0.109 ± 0.003	0.113 ± 0.004	0.396 ± 0.004	0.358 ± 0.008	0.176 ± 0.006	0.208 ± 0.007
**3**	*λ*_max_/nm	249 ± 0.5	249 ± 0.5	248 ± 1.0	248 ± 1.0	249 ± 0.5	249 ± 0.5
	*A*/a. u.	0.108 ± 0.006	0.113 ± 0.006	0.392 ± 0.006	0.368 ± 0.004	0.175 ± 0.005	0.203 ± 0.006
**avg**	*λ*_max_/nm	249 ± 0.5	249 ± 0.5	248 ± 1.0	248 ± 1.0	249 ± 0.5	249 ± 0.5
	*A*/a. u.	0.109 ± 0.006	0.112 ± 0.004	0.394 ± 0.005	0.362 ± 0.009	0.177 ± 0.006	0.205 ± 0.005
**RSD (%) of A**		5.5	3.6	1.3	2.5	3.4	2.4

**Table 2:** Model fish samples at different levels in the present study.

Fish Samples		Belonging to the Same
*Pampus argenteus* (PaA)	*Pseudaspius leptocephalus* (PL)	class
*Epinephelus rivulatus* (ER)	*Pagrosomus major* (PM)	order
*Scomberomorus niphonius* (SN)	*Scomber japonicus* (SJ)	family
*Larimichthys polyactis* (LP)	*Larimichthys crocea* (LC)	genus

## Data Availability

Data are contained within the article.

## Supplementary Materials

The following are available online. Comparison of muscle tissues on different parts of fish sample (S1); impact of dilution of initial protein extracts on distinguishing of fishes using UV-Vis spectrum (S2); comparison of the PCA scores for the UV-Vis spectra of different fish species (S3); distinguishing two fish species at different levels (S4); genetic test for the blind sample in the present study (S5); information about the fish samples used in the present study (S6).

## Sample Availability

Samples of the compounds are not available from the authors.
