# Supplementary material for: Rapid Identification between Two Fish Species Using UV-Vis Spectroscopy for Substitution Detection
Source: Molecules. 2021 Oct 28;26(21):6529. doi: 10.3390/molecules26216529 (PMC8587656; doi:10.3390/molecules26216529)
Supplement: Supplementary file 1 [file molecules-26-06529-s001.zip › molecules-1427759-supplementary.pdf]

Supplementary Materials for

Rapid identification between two fish species by UV-Vis spectroscopy for substitution detection

Zhaoliang Chai, Chengyu Wang, Hongyan Bi*

College of Food Science and Engineering, Shanghai Ocean University, Hucheng Ring Road 999, Pudong New District, 201306 Shanghai, China

*To whom correspondence should be addressed. Tel.: +86-21-61900364;

Fax: +86-21-61900365; E-mail address: hybi@shou.edu.cn

E-mail addresses for the other authors: chaizhaoliang2020@163.com; wangchengyu1002@163.com

S1. Comparison of muscle tissues on different parts of fish sample

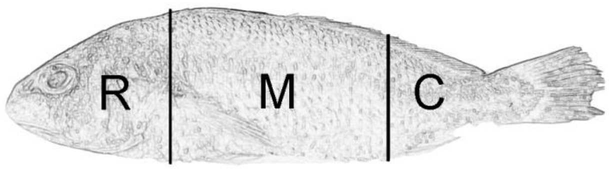

Figure S1. The three parts of the fish sample, including rostral (R), middle (M) and caudal (C) parts for sampling the muscle sample.

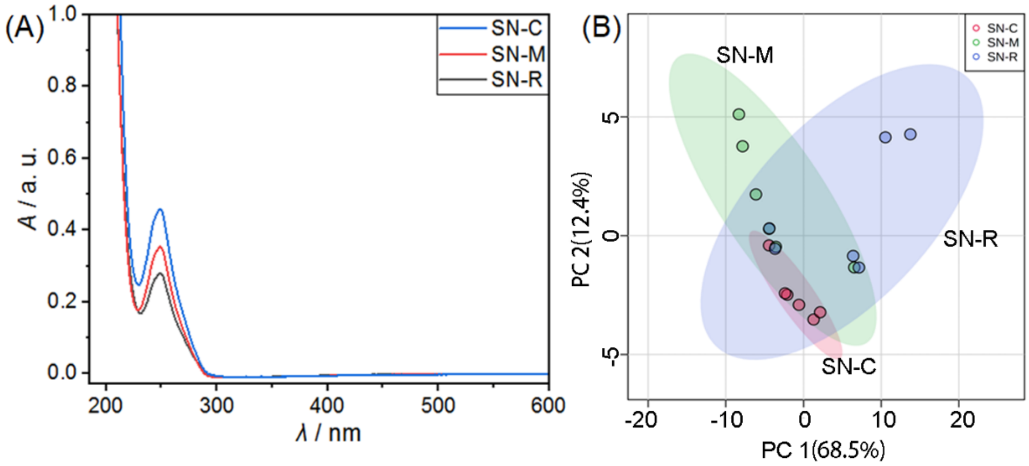

Figure S2. A) Representative UV-Vis spectra of solution of extracts from rostral (R), middle (M) and caudal (C) part of *Scomberomorus niphonius* (SN), respectively. B) Plot of PCA scores for UV-Vis spectra of three fish parts, that is, rostral (R), middle (M) and caudal (C) part of SN, respectively. Six UV-Vis spectra were obtained for each part. The initial extract of fish muscle tissue was diluted 80-fold with DI water.

S2. Impact of dilution of initial protein extracts on the distinguishment of fishes using UV-Vis spectrum

Figure S3 shows the plot of PCA scores for the UV-Vis spectroscopic results of different dilution folds (20, 40, 60, 80, 100) of the initial extracts of the analyzed fish species. It can be seen that the points for the the UV-Vis spectroscopic results under different dilution folds cluster in very close area, illustrating the distinguishment of the analyzed fish species under this data processing mode fails.

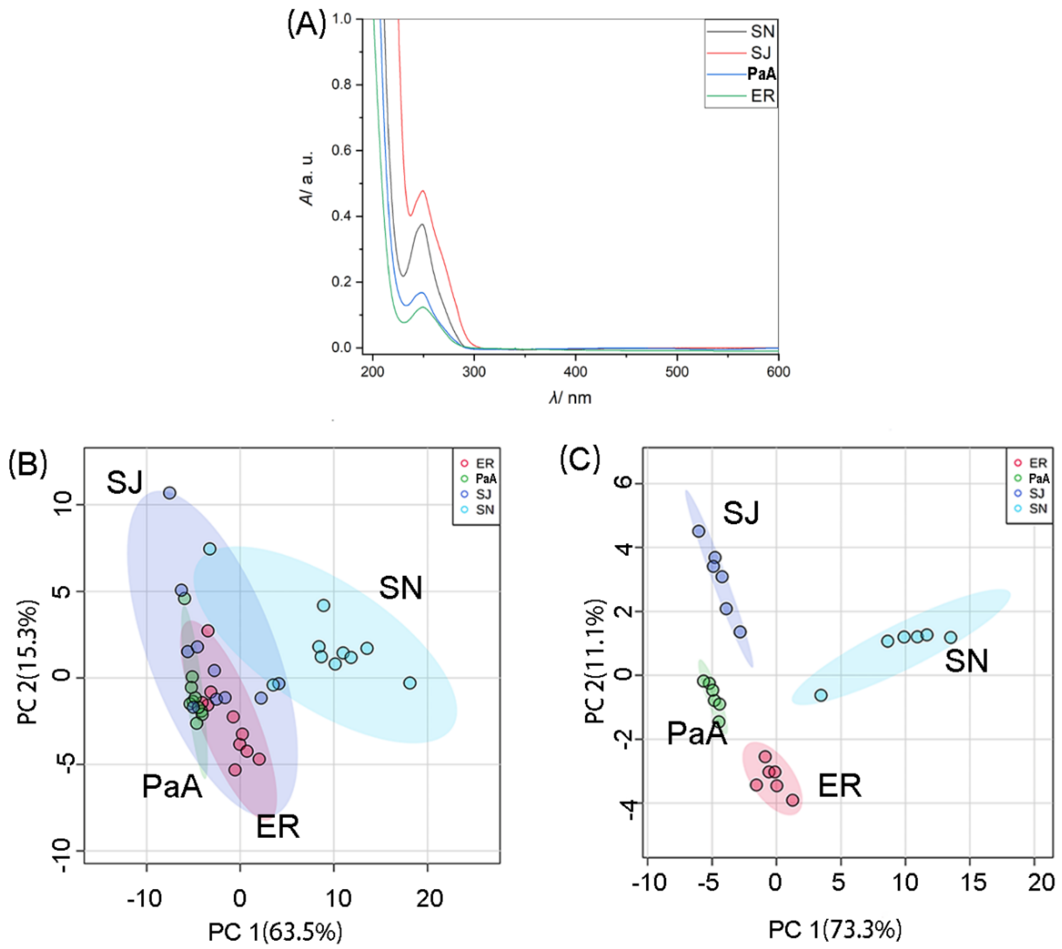

Figure S3. A) UV-Vis spectra of extracts from four fish samples of *Scomber japonicus* (SJ), *Scomberomorus niphonius* (SN), *Epinephelus rivulatus* (ER) and *Pampus argenteus* (PaA). B) Plot of principle component analysis (PCA) scores for the UV-Vis spectra of tissue extracts of the fish species SJ, SN, ER and PA. The obtained fish tissue extract was diluted with the fold of 20, 40, 60, 80, and 100, respectively. C) Plot of PCA scores of UV-Vis spectra of the four fish species. The obtained fish tissue extract was diluted 80-folds with DI water.

S3. Comparison of the PCA scores for the UV-Vis spectra of different fish species

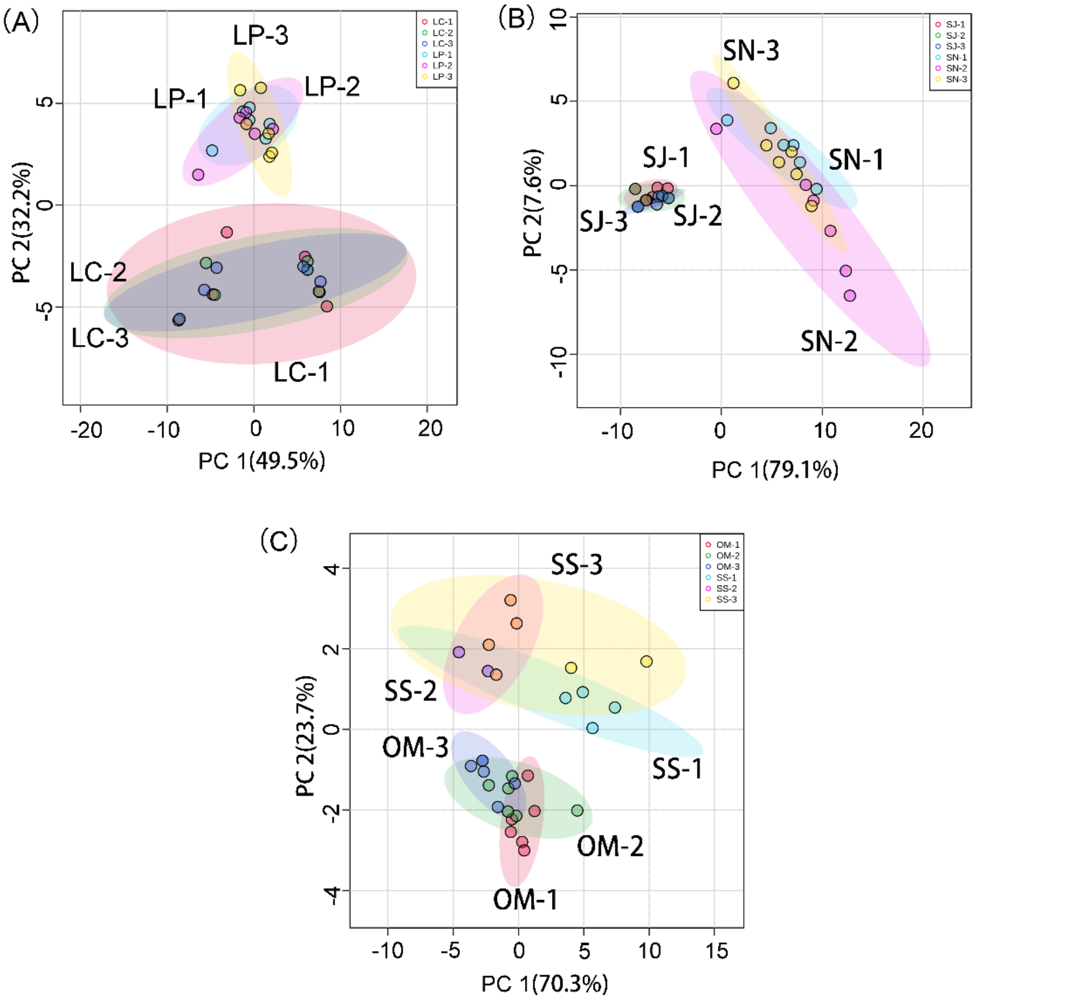

Figure S4. A) Plot of principal component analysis (PCA) scores for the UV-Vis spectra of fish species of *Larimichthys crocea* (LC) and *Larimichthys polyactis* (LP). B) Plot of PCA scores for the UV-Vis spectra of fish species of *Scomber japonicus* (SJ) and *Scomberomorus niphonius* (SN). C) Plot of PCA scores for the UV-Vis spectra of fish species of *Oncorhynchus mykiss* (OM) and *Salmo salar* (SS). The extraction was conducted following the protocol detailed in experimental section 3.2. The initial extract of fish muscle sample was diluted 80-fold with deionized (DI) water for UV-Vis spectroscopic analysis. Three runs of experiments were performed for each fish species. The UV-Vis spectra were collected from experiment run 1, 2 and 3.

S4. Distinguishment of two fish species at different levels

S4.1. Distinguishment of fish samples belonging to the same class

Epinephelus rivulatus (ER), *Scomber japonicus* (SJ), *Scomberomorus niphonius* (SN) and *Larimichthys crocea* (LP) belonging to the class Actinopteri were analyzed as model fishes belonging to the same class. The results shown in Figure S5 demonstrate that the two fish species belonging to the same class can be well distinguished.

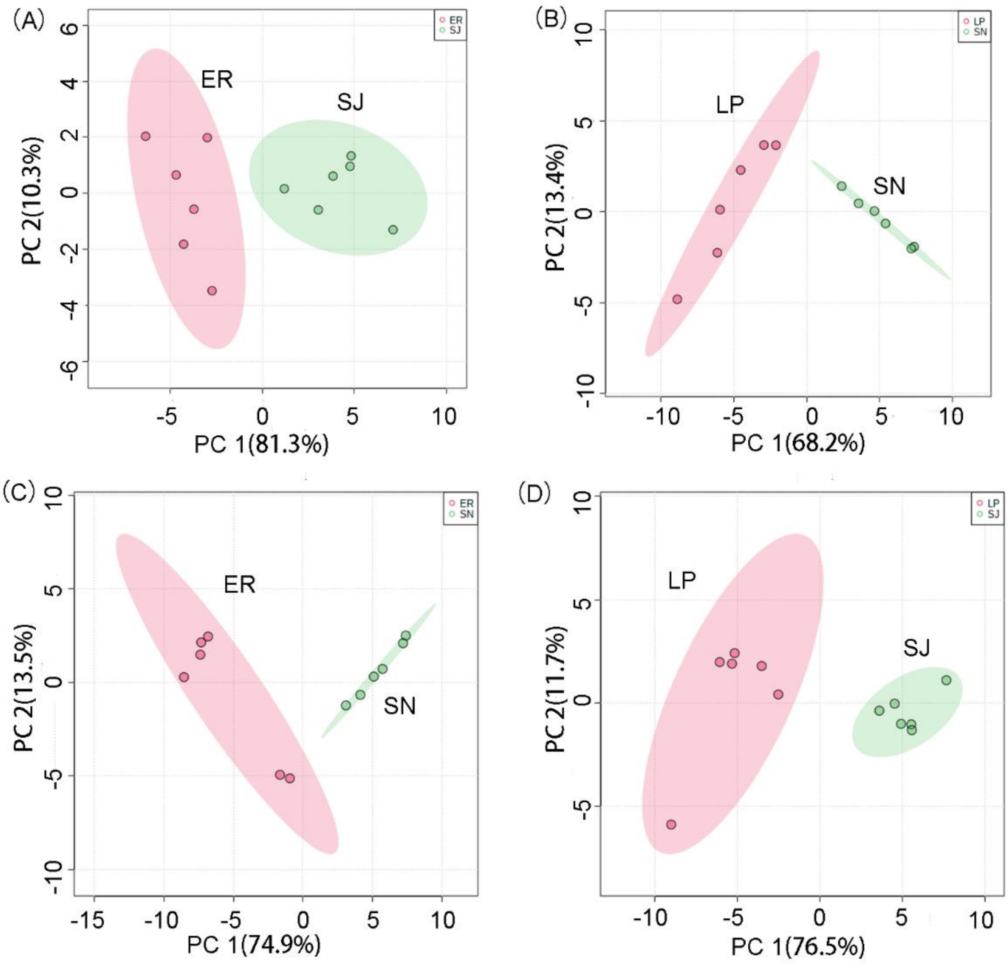

Figure S5. A) Plot of principle component analysis (PCA) score for the UV-Vis spectra of *Scomber japonicus* (SJ) and *Epinephelus rivulatus* (ER); B) Plot of PCA scores for the UV-Vis spectra of *Scomberomorus niphonius* (SN) and *Larimichthys crocea* (LP); C) Plot of PCA scores for the UV-Vis specta of *Scomberomorus niphonius* (SN) and *Epinephelus rivulatus* (ER); D) Plot of PCA scores for the UV-Vis spectra of *Scomber japonicus* (SJ) and *Larimichthys crocea* (LP).

S4.2. Distinguishment of fish samples belonging to the same order

Four fish species belonging to the order Perciformes were analyzed to verify the assumption in the main text, including *Larimichthys crocea* (LC), *Larimichthys polyactis* (LP), *Epinephelus rivulatus* (ER) and *Pagrosomus major* (PM). As shown in Figure S6, two fish species in an identical order can be well distinguished.

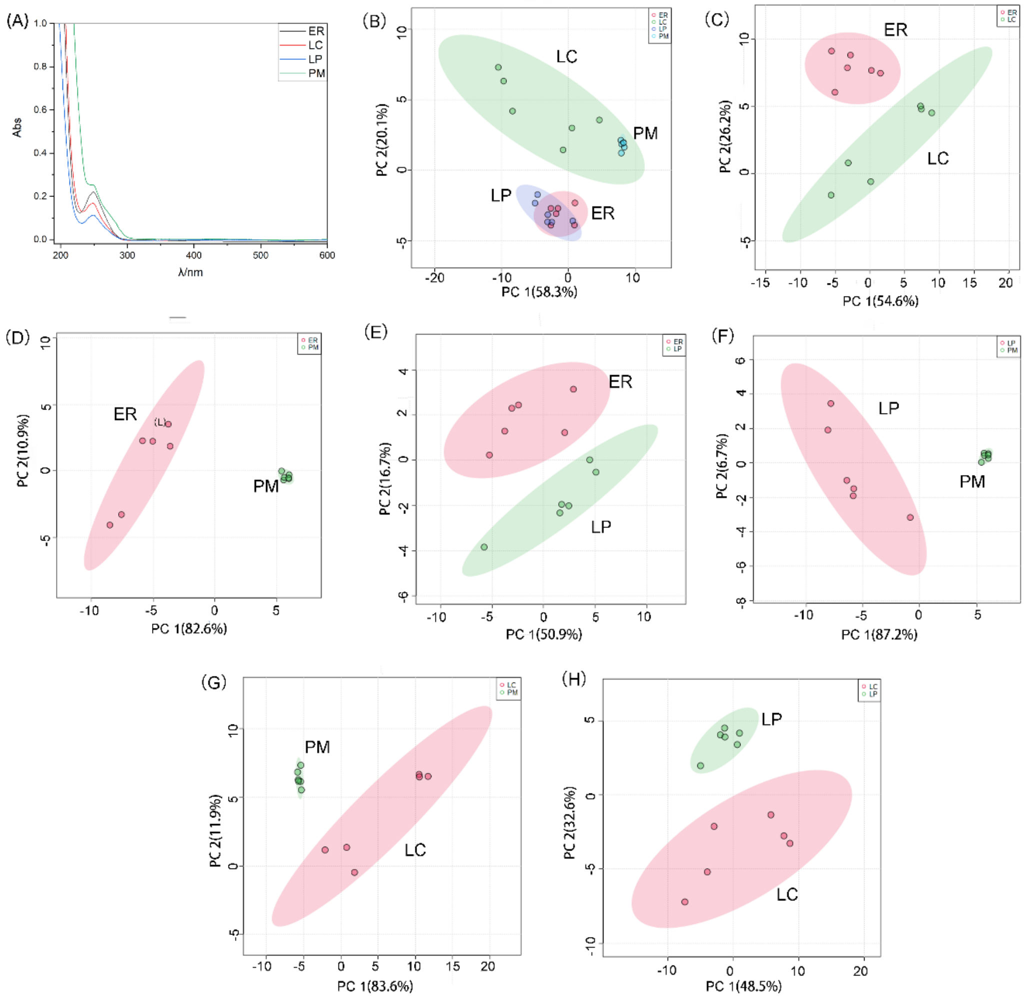

Figure S6. A) UV-Vis spectra of extracts from four fishes *Larimichthys crocea* (LC), *Larimichthys polyactis* (LP), *Epinephelus rivulatus* (ER) and *Pagrosomus major* (PM) belonging to an identical order. B) Plot of principle component analysis (PCA) scores for the UV-Vis spectra of four fish species. C) Plot of PCA scores for the UV-Vis spectra of ER and LC. D) Plot of PCA scores for the UV-Vis spectra of ER and PM. E) Plot of PCA scores for the UV-Vis spectra of ER and LP. F) Plot of PCA scores for

the UV-Vis spectra of LP and PM. G) Plot of PCA scores for the UV-Vis spectra of PM and LC. H) PCA score plot of LP and LC.

S4.3. Distinguishment of fish samples belonging to the same family

Scomber japonicus (SJ) and *Scomberomorus niphonius* (SN) belong to the same family Scombridae. *Culter alburnus* (CA) and *Pseudaspius leptocephalus* (PL) belong to the same family Cyprinidae. As shown in Figure S7, randomly chosen two fish species from the same family can be distinguished.

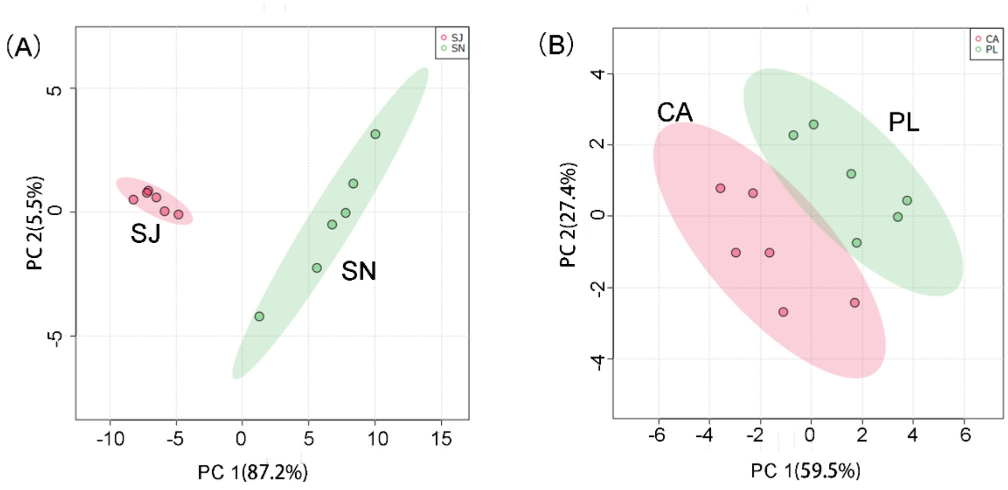

Figure S7. A) Plot of principle component analysis (PCA) scores for the UV-Vis spectra of *Scomber japonicus* (SJ) and *Scomberomorus niphonius* (SN). B) Plot of PCA scores for the UV-Vis spectra of *Culter alburnus* (CA) and *Pseudaspius leptocephalus* (PL).

S4.4. Distinguishment of fish samples belonging to the same genus

Larimichthys polyactis (LP) and *Larimichthys crocea* (LC) belong to the same genus Larimichthys. The results shown in Figure 4D show that two fish species from the same genus can be distinguished.

S5. Genetic test for the blind sample in the present study

Table S1. PCR reaction system.

ddH_2O	15.3 µL
10X Buffer	2 µL
template	1 µL
primer F	0.5 µL
primer R	0.5 µL
dNTP	0.5 µL
trans start Taq polymerase	0.2 µL
total volume	20 µL

Table S2. PCR procedure.

95 °C	5 min	
95 °C	30 s	
61 °C	30 s	10 cycles
72 °C	30 s	
95 °C	30 s	
58 °C	30 s	32 cycle
72 °C	30 s	
72 °C		
10 °C	5 min	

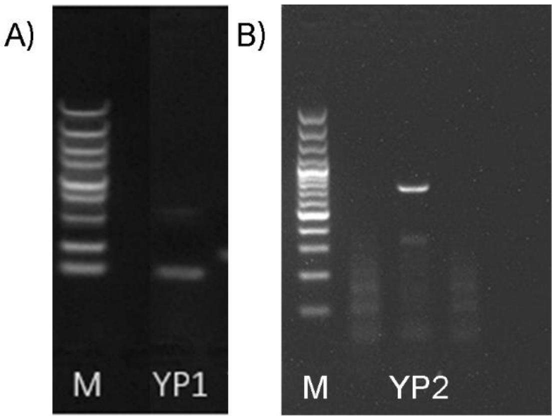

Figure S8. Electrophoresis results of polymerase chain reaction (PCR) of (A) unknown sample (US) in lane marked with YP 1 A), and sample from fish fillet of *Larimichthys Procea* as positive control in lane marked with YP 2 B). Lane marked with M is marker D2000 DNA Ladder (D2000).

Description	Common Name	Max Score	Total Score	Query Cover	E value	Per. Ident	Acc. Len	Accession
PREDICTED: Larimichthys crocea mitogen-activated protein kinase 7 (mapk7), transcript variant X2, mRNA	large yellow cr...	311	311	100%	9e-81	98.86%	3500	XM_027289425.1
PREDICTED: Larimichthys crocea mitogen-activated protein kinase 7 (mapk7), transcript variant X1, mRNA	large yellow cr...	311	311	100%	9e-81	98.86%	3701	XM_010751406.3
Larimichthys crocea extracellular signal-regulated kinase 5 (ERK5) mRNA, complete cds	large yellow cr...	311	311	100%	9e-81	98.86%	3720	KP343674.1
Larimichthys crocea genome assembly, chromosome: XIX	large yellow cr...	300	300	100%	2e-77	97.71%	20232972	LT972185.1

Figure S9. Sequence alignment results of gene amplification products of the unknown sample analyzed in this study.

Sequences producing significant alignments — Download — New Manage columns — Show 100

select all *100 sequences selected* — GenBank — Graphics — Distance tree of results — New MSA Viewer

Description	Scientific Name	Common Name	Taxid	Max Score	Total Score	Query Cover	E value	Per. Ident	Acc. Len	Accession
Larimichthys polyactis solate CO1B5 cytochrome oxidase subunit I (COI) gene, parti...	Larimichthys p...	yellow croaker	334908	610	610	100%	2e-170	97.77%	714	EU266386.1
Larimichthys polyactis mitochondrion, complete genome	Larimichthys p...	yellow croaker	334908	575	575	97%	9e-160	96.84%	16470	GU586227.1
Larimichthys polyactis mitochondrion, complete genome	Larimichthys p...	yellow croaker	334908	575	575	97%	9e-160	96.84%	16470	FJ618559.1
Larimichthys polyactis solate QD2014111901 cytochrome c oxidase subunit I (COX1)...	Larimichthys p...	yellow croaker	334908	569	569	93%	4e-158	97.62%	683	MF004322.1
Larimichthys polyactis solate QD2015012001 cytochrome c oxidase subunit I (COX1)...	Larimichthys p...	yellow croaker	334908	564	564	93%	2e-156	97.32%	684	MF004321.1
Larimichthys polyactis solate B15 cytochrome oxidase subunit I (COI) gene, partial c...	Larimichthys p...	yellow croaker	334908	564	564	94%	2e-156	97.05%	688	HM068248.1
Larimichthys polyactis solate B13 cytochrome oxidase subunit I (COI) gene, partial c...	Larimichthys p...	yellow croaker	334908	564	564	94%	2e-156	97.05%	688	HM068246.1
Larimichthys polyactis solate B9 cytochrome oxidase subunit I (COI) gene, partial cd...	Larimichthys p...	yellow croaker	334908	564	564	94%	2e-156	97.05%	688	HM068244.1
Larimichthys polyactis solate B5 cytochrome oxidase subunit I (COI) gene, partial cd...	Larimichthys p...	yellow croaker	334908	564	564	94%	2e-156	97.05%	688	HM068241.1

Figure S10. Sequence alignment results of gene amplification products of sample from fish fillet of *Larimichthys polyactis* analyzed in this study.

S6. Information about fish samples used in the present study

Table S3. Scientific classification of the seafood samples studied in the present work. The same classification levels are labeled with the same colors.

**fish species**	**class**	**order**	**family**	**genus**	**species**
Scomberomorus niphonius	Actinopteri	Scombriformes	Scombridae	Scomberomorus	*S. niphonius*
Scomber japonicus	Actinopteri	Scombriformes	Scombridae	Scomber	*S. japonicus*
Pampus argenteus	Actinopterygii	Scombriformes	Stromateidae	Pampus	*P. argenteus*
Epinephelus retouti	Actinopterygii	Perciformes	Serranidae	Epinephelus	*E. rivulatus*
Pagrosomus major	Actinopterygii	Perciformes	Sparidae	Pagrus	*P. major*
Larimichthys crocea	Actinopterygii	Perciformes	Sciaenidae	Larimichthys	*L. crocea*
Larimichthys polyactis	Actinopterygii	Perciformes	Sciaenidae	Larimichthys	*L. polyactis*
Culter alburnus	Actinopterygii	Cypriniformes	Cyprinidae	Culter	*C. alburnus*
Pseudaspius leptocephalus	Actinopterygii	Cypriniformes	Cypriniformes	Chanodichthys	*C. erythropterus*
Oncorhynchus mykiss	Actinopteri	Salmoniformes	Salmonidae	Oncorhynchus	*O. mykiss*
Salmo salar	Actinopteri	Salmoniformes	Salmonidae	Salmo	*S. salar*
Pennahia argentata	Actinopterygii	Perciformes	Sciaenidae	Pennahia	*P. argentata*

Table S4. Size and weight of fish samples analyzed in the present study. *Salmo salar* (SS) sample was obtained as fillet, and the size and weight of the whole fish samples are not available (N/A). Among the analyzed fish samples, SJ, SN, LC, LP, ER, PaA, PM, PeA and SS are marine fishes, while OM, CA and PL are freshwater fishes. The fishes SN, LC, LP, ER, PaA, OM, CA, PL, PeA and SS are predators, PM and SJ are omnivores.

**No.**	**species**	**trader**	**place of purchase (probable origin)**	**purchase time**	**biological replicates**	**length/cm**	**weight/g**
1	*Scomber japonicus* (SJ)	Sucheng raw food materials (苏呈食材)	Suzhou, Jiangsu, China	Aug. 2020	6	29.5±1.8	486.7±61.6
2	*Scomberomorus niphonius* (SN)	Sucheng raw food materials (苏呈食材)	Suzhou, Jiangsu, China	Aug. 2020	6	44.5±1.3	628.0±13.4
3	*Pampus argenteus* (PaA)	Yuge first class cabin (渔哥头等舱)	Qingdao, Shandong, China	Jul. 2020	6	15.5±0.4	60.7±2.3
4	*Epinephelus rivulatus* (ER)	Yuge first class cabin (渔哥头等舱)	Qingdao, Shandong province	Jul. 2020	3	42.0±9.8	703.3±46.7
5	*Culter alburnus* (CA)	Qiandao Yuxiang (千岛鱼香)	Hangzhou, Zhejiang, China	Aug. 2020	6	26.5±2.6	108.3±26.7
6	*Pseudaspius leptocephalus* (PL)	Qiandao yuxiang (千岛鱼香)	Hangzhou, Zhejiang, China	Aug. 2020	6	34.9±1.8	262.5±42.5
7	*Larimichthys crocea* (LC)	Jinxiansheng aquatic products (金先生海产)	Ningbo, Zhejiang, China	Aug. 2020	6	26.6±5.8	317.5±17.5
8	*Pagrosomus major* (PM)	Siji seafood store (四季海鲜店)	Yantai, Shandong, China	Aug. 2020	6	28.4±2.9	412.5±67.5
9	*Larimichthys polyactis* (LP)	Yuge first class cabin (渔哥头等舱)	Qingdao, Shandong, China	Aug. 2020	6	17.7±1.2	67.5±7.5
10	*Pennahia argentata* (PeA)	Shili (Ten Mile) Seafood (十里海鲜店)	Zhanjiang, Guangdong, China	Dec. 2020	6	17.5±1.8	55.5±8.5
11	Salmo salar (SS)	Ftabest (Lingang, Shanghai) (自贸优选)	Shanghai, China	Dec. 2020	3 fillets	N/A	N/A
12	Oncorhynchus mykiss (OM)	Yinggui Plantation business (应桂农园商贸)	Qingdao, Shandong, China	Dec. 2020	3	40.5±7.5	805.6±215.2
